# Multiple genome comparison based on overlap regions of pairwise local alignments

**DOI:** 10.1186/1471-2105-13-S19-S7

**Published:** 2012-12-19

**Authors:** Katharina Jahn, Henner Sudek, Jens Stoye

**Affiliations:** 1 Department of Mathematics and Statistics, University of Ottawa, Canada; 2 Technische Fakultät, Universität Bielefeld, Germany

## Abstract

**Background:**

Mancheron, Uricaru and Rivals (*Nucleic Acids Res*. 39:e101, 2011) recently introduced a new approach in the context of multiple genome comparison that allows to detect regions of strong overlaps in a set of pairwise local alignments between several reference genomes and one target genome. Such overlap regions are an important source of information in genome annotation.

**Results:**

In this paper we introduce a series of algorithms that improve over the approach of Mancheron *et al*., both in terms of computational complexity and in practical runtime. We also extend the problem definition such that overlaps to different reference genomes can be rated differently and regions overlapping only a subset of the reference genomes are detected.

## Background

Comparative approaches are an important source of information when it comes to the analysis of newly sequenced genomes. On the level of genes, the use of reciprocal BLAST hits is the most widely accepted approach suitable for tasks like gene annotation and the inference of homologies. However, it is a notoriously slow process, especially when it comes to all-against-all comparisons of several genomes, as commonly used in multiple genome comparisons. The alignment of whole genomes is known to be a computationally hard problem [1]. Heuristic approaches such as Shuffle-LAGAN [2] or MAUVE [3] involve a combination of chaining local alignments and inferring the history of large-scale genome rearrangement processes, which is an NP-hard problem of its own [1].

Recently, Mancheron *et al. *[4] pointed out that for many goals in multiple genome comparison, such as gene annotation, whole genome-based approaches as well as all-against-all BLAST comparisons are too involved. Instead they suggest to identify regions of strong overlaps in a set of pairwise local alignments between one target genome and any number of reference genomes. A strong overlap is a region that maps to at least one segment in every reference genome. If it is not contained in a bigger region that fulfills the same property, it is called a *maximum common interval *(MCI). In the same paper, Mancheron *et al*. present an algorithm to compute all MCIs for *k *genomes and a total number of *n *mappings in *O*(*n *log *k*) time and *O*(*n*) space. Note that the term "common interval" has been used previously in a different context, in order to describe a set of genes that occur as a consecutive block in two or more genomes [5,6]. To avoid confusion of the two concepts, we use the term *maximum overlapping interval *(MOI) in this paper when referring to the concept of *maximum common interval *by Mancheron *et al*.

In this paper, we re-visit the above problem and introduce three new algorithms that improve the asymptotic time complexity as well as the practical performance over the approach of Mancheron *et al. *[4]. At first we present an algorithm that requires *O*(*n*) time and space. Then we show two variations of this basic algorithm: While the first modification reduces memory consumption but keeps the *O*(*n*) running time, the second one gives up on linear worst case runtime, but seems to have linear average runtime and is very fast in practice. We also introduce a generalization of the MOI problem, which includes the case where an MOI may map to only *q *out of *k *reference genomes. We show how the first two of our algorithms can be adapted to this variant.

## Methods

### Preliminaries

For the basic definitions of maximum overlapping intervals we follow closely the notation of Mancheron *et al. *[4]. Given a finite sequence *S*, let *|S| *denote its length. An interval *I *of a sequence *S *is a pair *I *= [*s*(*I*), *e*(*I*)] of start and end positions within *S*, i.e. 1 ≤ *s*(*I*) ≤ *e*(*I*) ≤ *|S|*. The length of *I *is defined as *e*(*I*) - *s*(*I*) + 1. The subset relation is defined as usual for intervals, i.e. *I*_1 _⊆ *I*_2 _if and only if *s*(*I*_2_) ≤ *s*(*I*_1_) and *e*(*I*_1_) ≤ *e*(*I*_2_).

In the following a target genome is given as a sequence *T *together with *k *reference genomes *G*_1_, . . ., *G_k_*. For each *j*, 1 ≤ *j *≤ *k*, let $C_{j}=\left(I_{1}^{j},I_{2}^{j},...,I_{n_{j}}^{j}\right)$ be the collection of *base intervals *representing mappings of *G_j _*to *T*.

An interval *J *is an *overlapping interval *of a set of collections *C *= {*C*_1_*, . . ., C_k_*} if and only if there exists an interval $I_{i}^{j}$ in every *C_j _*with $J\subseteq I_{i}^{j}$. An overlapping interval *J *= [*p*, *q*] with *p *≤ *q *is *maximum *if neither [*p *- 1, *q*] nor [*p*, *q *+ 1] are overlapping intervals.

The computational problem we study in this paper is the following: given a set of collections of base intervals, find all its maximum overlapping intervals (MOIs).

### Upper bound for the number of MOIs

Before presenting our algorithms, we derive a tight upper bound for the number of MOIs in a set of *k *collections with a total number of *n *base intervals.

As already shown in supplementary file 1 of [4], asymptotically there can only be *O*(*n*) MOIs: every MOI starts at the beginning of a base interval and no MOI can be included in another MOI, which means that no two MOIs can have the same starting point, so clearly there can not be more than *n *MOIs. In fact, the bound can be given more precisely:

**Lemma 1**. *For k collections with a total number of n base intervals, there can be at most n* - *k *+ 1 *maximum overlapping intervals. This is a tight bound*.

***Proof***. Assume the MOIs to be ordered by their beginning from left to right. Clearly, the leftmost MOI must contain at least *k *base intervals. Moreover, moving to the right, the next MOI requires at least one new interval, otherwise it would be identical to the previous one. This argument can be repeated for all remaining MOIs. Since after the first MOI there are only *n *- *k *intervals left, the total number of MOIs can not be larger than 1 + *n *- *k*.

**Figure 1:**
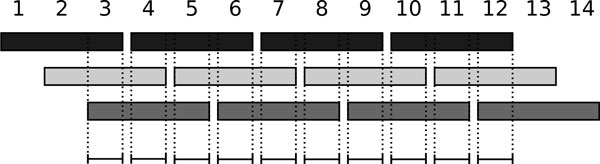
**Interval collections with a maximized number of MOIs**. Example of interval collections with a maximized number of MOIs. The bars indicate the location of the *n *= 12 base intervals in *T*, their colors define the distribution into *k *= 3 collections (dark, light, medium gray). MOIs are drawn as black lines at the bottom. Their number equals the upper bound *n* - *k *+ 1 = 10.

To show that the bound is tight, we construct an example where the number of MOIs is actually *n *- *k *+ 1. We set the length of all base intervals to *k*. The first base interval of the *j*th collection starts at index *j*, and the base intervals within each collection follow one after the other such that the *i*th interval of the *j*th collection starts at index *j *+ *k*(*i *- 1). By doing this, the intersection of the first *k *base intervals defines the first MOI. After that we can replace the left-most starting interval by the next one from its collection. The intersection of the new interval set gives us the next MOI. This can be repeated *n *- *k *times until all intervals have been processed. Figure 1 illustrates this construction.   □

### Algorithms for finding maximum overlapping intervals

In this section we present three new algorithms to find maximum overlapping intervals. Without loss of generality we assume that the set of base intervals is *compact*, meaning that at least one interval starts or ends at every position in *T *and thus *l *:= *|T | *∈ *O*(*n*). We also assume that the list of base intervals is already sorted by increasing start positions. This can be done in linear time using the radix sort algorithm.

#### Algorithm LinearMOI

The outline of our first algorithm, LinearMOI, is as follows: while going through the sorted list of base intervals, we track for each of the *k *collections the largest end point of any of its intervals processed so far. Once we have processed all intervals with the same start position, we test whether the smallest of the current end points, denoted as *min*, is smaller than the recent start position. If so, there is at least one collection that does not cover the start position, and there is no MOI. If *min *is greater than the recent start position, then the segment in between is covered by all collections, and is therefore part of an MOI. We only need to test if we have already used *min *as end point for an MOI starting further to the left. Otherwise the interval ranging from the recent start position to *min *is an MOI.

Obviously, *min *can change with each new base interval. To update it efficiently, we use a counter array *c*, indexed from 0 to *l*, where *l *is the length of the target genome. In this array we store at each position the number of collections that have their current end point at this position. Clearly, *min *equals the index of the smallest non-zero entry. The following observation helps us to track this position.

**Observation 1**. *Values in c at indices smaller than the current leftmost non-zero entry do not change when a new base interval is considered*.

Using Observation 1 we can find the current leftmost non-zero entry in *c *simply by starting from the former leftmost non-zero entry and going to the right until we encounter the first non-zero value. Although we may need several steps for one update of *min*, the total number of steps can not be larger than *l*, as *min *is never decreased. As all other operations for processing a base interval take constant time, and *c *and *endPoint *have at most *n *entries, we get a total runtime of *O*(*l*). By our assumption of compactness we thus have a runtime linear in the number of base intervals *O*(*n*).

**Figure 2:**
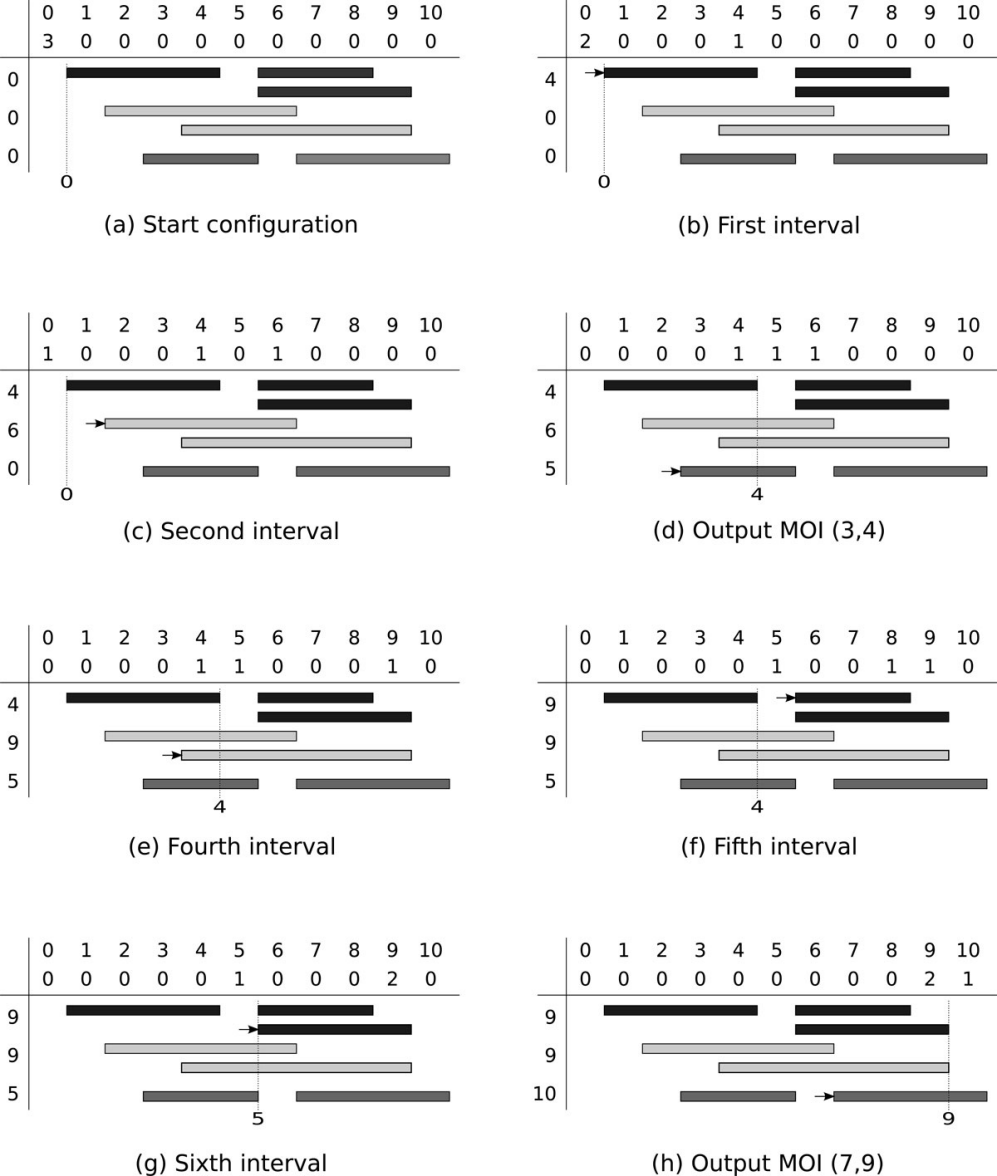
**Example of algorithm LinearMOI**. Example of algorithm LinearMOI with *C*_1 _= {(1, 4), (6, 8), (6, 9)} (dark gray), *C*_2 _= {(2, 6), (4, 9)} (light gray) and *C*_3 _= {(3, 5), (7, 10)} (medium gray). The left column shows the state of *endPoint*, *c *is shown above the intervals, and *min *is represented by the dotted vertical line. The arrow points to the interval currently processed. Output is (3, 4) in step (d) and (7, 9) in step (h).

Pseudocode of the algorithm is shown in Algorithm 1 (LinearMOI). An example with *k *= 3 collections and *n *= 7 base intervals is shown in Figure 2.

**Table 1:** 

Algorithm 1 (LinearMOI)
**Input: **sorted list of all intervals *interval*[1*..n*]; number of collections *k *
**Variables: **largest end point seen so far in each collection *endPoint*[1*..k*]; *c*[0*..l*]
1: *endPoint*[1*..k*] *← *0
2: *prevEnd ← *0
3: *min ← *0
4: *c*[0] *← k*; *c*[1*..l*] *← *0
5: **for all $\left(I_{i}^{j}=\left[start,\;end\right]\right)$ ∈ ***interval*[1*..n*] **do **
6: **if ***end *>*endPoint*[*j*] **then **
7: *c*[*endPoint*[*j*]] *← c*[*endPoint*[*j*]] - 1
8: *c*[*end*] *← c*[*end*] + 1
9: *endPoint*[*j*] *← end *
10: **end if **
11: **if **all intervals with recent start position processed **then **
12: **while ***c*[*min*] = 0 **do **
13: *min ← min *+ 1
14: **end while **
15: **if ***prevEnd < min ***and ***min ≥ start ***then **
16: output MOI(*start*, *min*)
17: *prevEnd ← min *
18: **end if **
19: **end if **
20: **end for **

#### Algorithm CircularMOI

The space needed to store the counter array *c *of algorithm LinearMOI may become problematic when large genomes are processed. Assuming the human genome as our target, an additional 4GB of memory would be required. However, we find that only a small part of *c *is informative in each step of the algorithm:

**Observation 2**. *We only need to know if there is any non-zero entry in c left of the recent start position and not where or what this value is*.

**Observation 3**. *All positions in c that are further to the right of the recent start position than the length of the longest base interval have value *0.

Based on these observations we can replace array *c *by a circular memory structure that is just as long as the longest base interval as well as a single counter in which we store the sum of all values that would be on the left of our current position in the counter array.

To employ the new data structure, several modifications need to be made in algorithm LinearMOI. Obviously we need to use the modulo operation whenever accessing *c*. We also have to make sure that we do not update *c *at indices smaller than the recent start position as these positions are no longer represented in the array. For that purpose, we introduce a new variable *open *to count the number of collections that cover the recent start position.

To keep *open *updated, we change the role of variable *min*: when processing a new interval, we increase *min *until the recent start position is reached. Any non-zero value encountered in this process is subtracted from *open *and then set back to 0, so that the respective array positions can be re-used. We also need to update *open *whenever a collection gets a new endpoint and is not already counted in *open*.

Finally, we know that we can only have an MOI if *open *equals *k*. Therefore we add a test for this condition in line 11. Note that if this condition holds, we use *min *in its original role to find the endpoint of a potential MOI. The final test before reporting an MOI reduces to testing whether *prevEnd *is smaller than *min*. The complete pseudocode of this algorithm is shown in Algorithm 2 (CircularMOI).

In practice this approach is quite slow because of the extensive use of the modulo operation. However when extending the length of *c *to the next power of two, the modulo operation can be replaced by a very fast bitwise AND operation, and one gets about the same performance as in the first algorithm. This variant of algorithm CircularMOI was used in the benchmark tests described in the Results section.

#### Algorithm TestMOI

Having concentrated on asymptotic runtime so far, we now focus on practical performance. Even though the previous algorithms perform very well in our benchmark experiments, as we will demonstrate in the Results section they use linear extra memory which might limit their usability for larger datasets.

**Table 2:** 

Algorithm 2 (CircularMOI)
**Input: sorted list of intervals *interval*[1*..n*]; number of collections *k*; length of the longest interval *ℓ***
**Variables: largest end point seen so far in each collection *endPoint*[1*..k*]; *c*[0*..l*]**
1: *endPoint*[1*..k*] *← *0
2: *prevEnd ← *0
3: *min ← *0
4: *c*[0*..ℓ*] *← *0
5: *open ← *0
6: **for all **$\left(I_{i}^{j}=\left[start,\;end\right]\right)$**∈ ***interval*[1*..n*] **do **
7: **while ***min < start ***do **
8: *open ← open - c*[*min *mod *ℓ*]
9: *c*[*min *mod *ℓ*] *← *0
10: *min ← min *+ 1
11: **end while **
12: **if ***end > endPoint*[*j*] **then **
13: **if ***endPoint*[*j*] *≥ min ***then **
14: *c*[*endPoint*[*j*] mod *ℓ*] *← c*[*endPoint*[*j*] mod *ℓ*] - 1
15: **else **
16: *open ← open *+ 1
17: **end if **
18: *c*[*end *mod *ℓ*] *← c*[*end *mod *ℓ*] + 1
19: *endPoint*[*j*] *← end *
20: **end if **
21: **if **all intervals with recent start position processed **and ***open *= *k ***then **
22: **while ***c*[*min *mod *ℓ*] = 0 **do **
23: *min ← min *+ 1
24: **end while **
25: **if ***prevEnd < min ***then **
26: output MOI(*start*, *min*)
27: *prevEnd ← min *
28: **end if **
29: **end if **
30: **end for **

We present a third algorithm, TestMOI, that works without a counter array. To find *min*, it just takes the minimum stored in *endPoint*. Finding this smallest value takes *O*(*k*) time and might be needed for every interval, so we get a total runtime of *O*(*kn*). Using the following observation as a runtime heuristic, we get back to near-linear performance.

**Observation 4**. *The minimum value of a set of numbers can only increase if a value equal to the smallest value in the set is removed or increased*.

Therefore a test for a new minimum needs to be performed only when the smallest of the current endpoints changes. This gives rise to the procedure shown in Algorithm 3 (TestMOI).

Assuming the endpoints of the intervals are randomly distributed, the chance of two or more values in *endPoint *to be identical is very small and the chance that an interval is in the same collection as the smallest current value is 1*/k*. According to this the expected runtime would be *O*(*kn · *1*/k*) = *O*(*n*). However, this model is possibly oversimplified as there surely is a correlation between the start and the endpoint of an interval, as the base intervals are not the result of a random process but come from local alignments between the target and the reference genomes. Nevertheless the benchmark experiments in the Results section show that in practice the behavior is indeed linear.

**Table 3:** 

Algorithm 3 (TestMOI)
**Input: sorted list of all intervals *interval*[1*..n*]; number of collections *k***
**Variables: largest end point seen so far in each collection *endPoint*[1*..k*]**
1: *endPoint*[1*..k*] *← *0
2: *prevEnd ← *0
3: *min ← *0
4: *newEndPoint ← false *
5: **for **$\left(I_{i}^{j}=\left[start,\;end\right]\right)$**∈ ***interval*[1*..n*] **do **
6: **if ***end *>*endPoint*[*j*] **then **
7: **if ***endPoint*[*j*] = *min ***then **
8: *newEndPoint ← true *
9: **end if **
10: *endPoint*[*j*] *← end *
11: **end if **
12: **if ***newEndPoint ***and **all intervals with recent start position processed **then**
13: *min ← min*_*i *= 1..*k*_{*endPoint*[*i*]}
14: *newEndPoint ← false *
15: **if ***prevEnd < min ***and ***min ≥ start ***then **
16: output MOI(*start*, *min*)
17: *prevEnd *= *min *
18: **end if **
19: **end if **
20: **end for **

### Weighted maximum overlapping intervals

We now introduce the concept of *weighted *maximum overlapping intervals. One constraint for MOIs was that there had to be at least one interval in every collection that covers the MOI. For weighted MOIs we replace this constraint by the following: we assign a positive integer weight to every collection, and require the sum of the weights of all collections covering a weighted MOI to be at least as high as a predefined threshold *w*. The formal definitions are as follows:

Given a positive integer weight *w*, an interval *J *is a *w-overlapping interval *of a set of collections *C *= {*C*_1_, . . ., *C_k_*} if and only if the sum of weights of all collections *C_j _*that contain an interval $I_{i}^{j}$ with $J\subseteq I_{i}^{j}$ is at least *w*.

A *w*-overlapping interval *J *= [*p, q*] with *p *≤ *q *is *maximum *if neither [*p *- 1*, q*] nor [*p, q *+ 1] are *w*-overlapping intervals.

**Figure 3:**
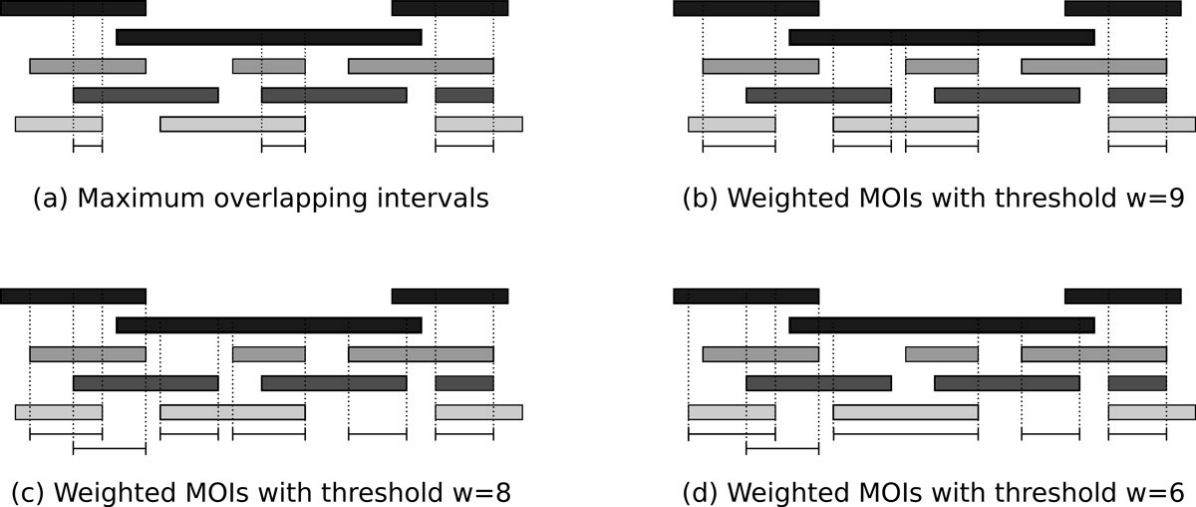
**Example of weighted MOIs**. Example for weighted MOIs in four collections (distinguished by different gray shadings). The weights of the collections are *w*(*C*_1_) = 2, *w*(*C*_2_) = 3, *w*(*C*_3_) = 3 and *w*(*C*_4_) = 4.

We refer to maximal *w*-overlapping intervals as weighted MOIs. Note that this concept subsumes the natural extension of MOIs by a quorum parameter *q*, where an interval is considered an MOI whenever it is covered by base intervals from at least *q *≤ *k *collections. We only need to assign weight 1 to every collection and set the threshold to *w *= *q*. By choosing appropriate weights, weighted MOIs allow us to solve more general problems like finding all MOIs that must have an interval in *C*_1 _and one interval either in *C*_2 _or in *C*_3_. An example of weighted MOIs for different thresholds is given in Figure 3. Our first two algorithms LinearMOI and CircularMOI can be adapted straightforwardly to weighted overlapping intervals as shown in the following.

#### Algorithm LinearWeightedMOI

This algorithm follows the same ideas as Algorithm 1 (LinearMOI) but we have to make several small adjustments. First we need to change the counter array such that it stores the weights of the collections. The second modification is a bit more complicated. In the unweighted case all values in *c *left of *min *are 0, which is not true in the weighted case. To deal with this, we use a similar technique as we already used in Algorithm 2 (CircularMOI). But instead of counting how many collections cover the recent start position, we count the sum of their weights in a variable named *openWeight*.

In the beginning, *openWeight *is set to zero. When updating *min *or *c*, we have to make sure that *openWeight *gets updated appropriately, which we do in lines 10-12 and 17 in Algorithm 4 (LinearWeightedMOI). We increase *min *(line 18), as long as *openWeight *exceeds *minWeight*. If *min *was increased since the last weighted MOI was reported and is not smaller than the recent start position, we have found a new weighted MOI.

**Table 4:** 

Algorithm 4 (LinearWeightedMOI)
**Input: sorted list of intervals *interval*[1*..n*]; number of collections *k*; weight of the collections *weight*[1*..k*]; length *l *of the target genome; minimum weight a weighted MOI must have *minWeight***
**Variables: largest end point seen so far in each collection *endPoint*[1*..k*]; *c*[0*..l*]**
1: *endPoint*[1*..k*] *← *0
2: *prevEnd ← *0
3: *min ← *0
4: *openWeight ← *0
5: *c*[0*..l*] *← *0
6: **for all **$\left(I_{i}^{j}=\left[start,\;end\right]\right)$**∈ ***interval*[1*..n*] **do **
7: **if ***end *>*endPoint*[*j*] **then **
8: *c*[*endPoint*[*j*]] *← c*[*endPoint*[*j*]] - *weight*[*j*]
9: *c*[*end*] *← c*[*end*] + *weight*[*j*]
10: **if ***endPoint*[*j*] * < min *and *end *≥ *min ***then **
11: *openWeight ← openWeight *+ *weight*[*j*]
12: **end if **
13: *endPoint*[*j*] = *end *
14: **end if **
15: **if **all intervals with recent start position processed **then **
16: **while ***openWeight * - *c*[*min*] *≥ minWeight ***do **
17: *openWeight ← openWeight - c*[*min*]
18: *min ← min *+ 1
19: **end while **
20: **if ***prevEnd < min ***and ***min ≥ start ***then **
21: output MOI(*start*, *min*)
22: *prevEnd ← min *
23: **end if **
24: **end if **
25: **end for **

#### Algorithm CircularWeightedMOI

In order to adapt Algorithm 4 (LinearWeightedMOI) to using the circular memory structure, we basically follow the same strategy as we did in the unweighted case. However there is no need to introduce an additional variable as was done for Algorithm 2. This is because we already use variable *openWeight *which takes over the function of *open *in Algorithm 2. Apart from this, there is only one non-trivial change in comparison to the unweighted case: a second condition in the **if **statement in line 12 to ensure that the endpoint of the current base interval is not smaller than the current *min*. This is because in the weighted case the current base interval can end to the left of the current location of *min*, and we have to make sure not to update *c *at indices smaller than *min*. The details are left to the reader.

## Results and discussion

To analyze the practical run times of our algorithms, we implemented them in C++ and compared them to the original implementation of the approach by Mancheron *et al. *[4] which is part of the QOD-1.0.0 software package and available under the CeCILL software license.

All benchmarks were performed using an Intel(R) Core(TM)2 Duo CPU T8100 @ 2.10GHz processor with 4GB RAM running Linux 3.3.4. We used the gcc version 4.7 with compiler flags -Ofast and -march = nativeset. If no other values are given, the length of the target genome is 10 Mb and the length of the base intervals is normally distributed around a mean of *μ *= 2 Kb with a standard deviation of σ = 0.5*μ*. A total of 5 million intervals are distributed randomly into 200 collections. In order to compare the weighted and unweighted algorithms we set all weights to 1 and the threshold *minWeight *to *k*. Please note that the time for sorting the input is not included in any of the reported timings. In any case, sorting the input will virtually take the same time for all algorithms, even though QOD sorts every collection separately, while we sort all base intervals in a single list.

**Figure 4:**
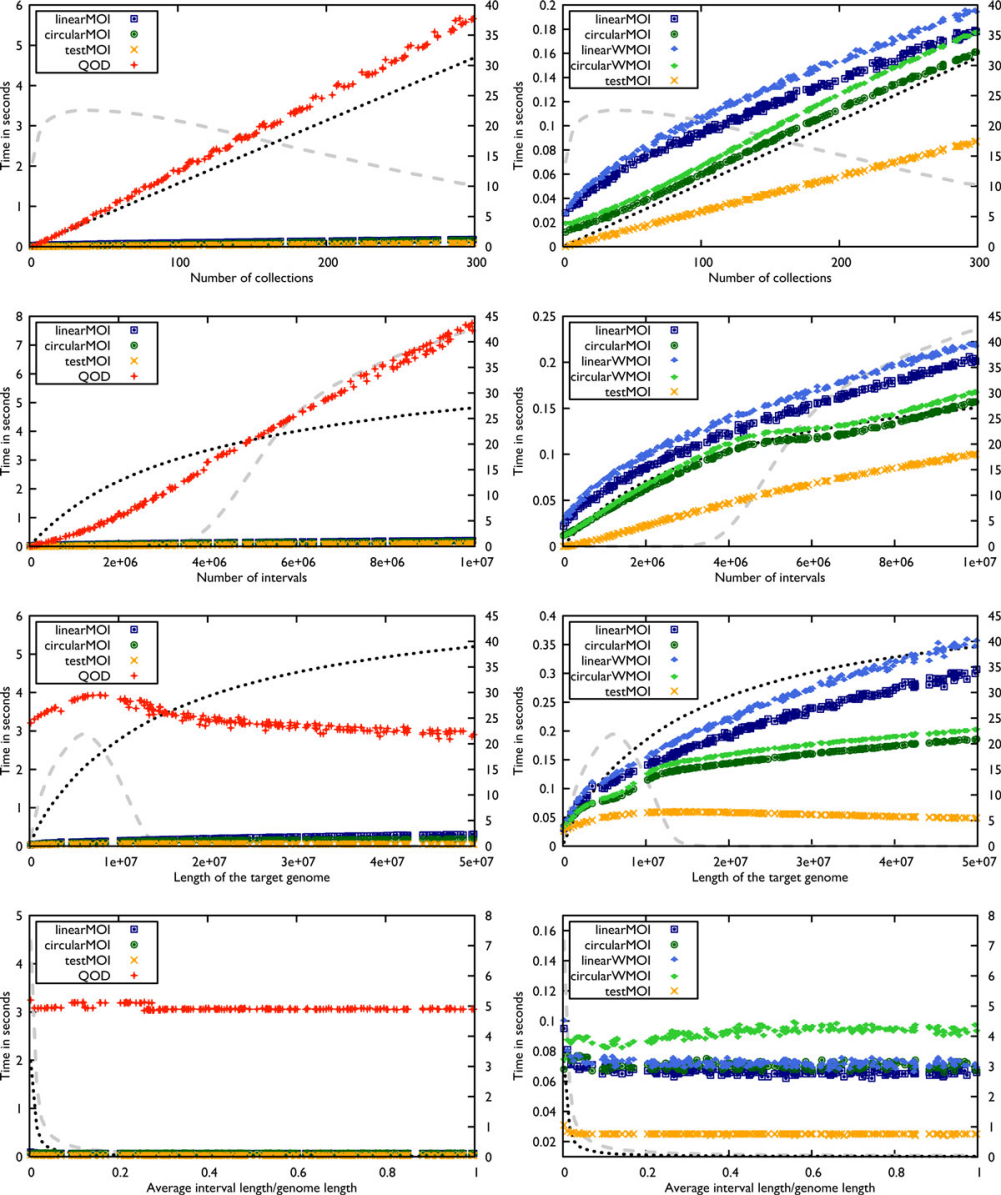
**Benchmark experiments**. Dependency of practical runtimes on parameter settings. First line: number of collections, on average 25.000 intervals per collection; Second line: number of intervals, fixed number of collections; Third line: length of target genome, fixed number of collections and intervals; Fourth line: average interval length relative to genome length for collections generated with *μ *between 1 and the genome length. The dotted line shows the number of non-redundant base intervals (scaled by secondary *y*-axis times 10^5^); the dashed line shows the number of MOIs (scaled by secondary *y*-axis times 10^3^).

We ran several tests in order to evaluate the performance of the algorithms for various parameter settings. The results are shown in Figure 4. In all tests our algorithms outperformed the algorithm used by QOD, and TestMOI is the fastest of all.

## Conclusions

In this paper we studied an algorithmic problem that was recently introduced by Mancheron *et al. *[4] in the context of multiple genome comparison. The goal is to find regions of strong overlaps in a set of pairwise local similarities between several reference genomes and one target genome.

We have presented efficient algorithms to solve this problem, two of which have asymptotically optimal, linear runtime. The third one excelled in terms of practical performance. All three algorithms were shown to outperform the approach introduced by Mancheron *et al. *[4]. We have also generalized the problem such that segments in overlap regions can be scored differently based on the reference genome they originate from. We have shown how this problem can still be solved in linear time.

For further work it may be interesting to assign individual weights to the base intervals. However we would then have to consider also intervals that are nested into other intervals of lower weight and therefore lose the strict-linear ordering for processing the intervals. Hence, we can not expect that the algorithms we presented here will be easily adaptable to this problem and still run in linear time.

## Authors' contributions

KJ, HS and JS jointly developed the algorithms for the unweighted MOI problem. HS developed the algorithm for the weighted case and carried out the computational studies. KJ and JS drafted the manuscript. All authors read and approved the final manuscript.

## Competing interests

The authors declare that they have no competing interests.
